# Artificial macropores improve maize performance at the seedling stage under poor aeration

**DOI:** 10.3389/fpls.2024.1468242

**Published:** 2024-10-03

**Authors:** Peng Xiong, Fahui Jiang, Yuekai Wang, Zhongbin Zhang

**Affiliations:** ^1^ Key Laboratory of Agricultural Resources and Ecology in Poyang Lake Watershed of Ministry of Agriculture and Rural Affairs in China, College of Land Resource and Environment, Jiangxi Agricultural University, Nanchang, China; ^2^ State Key Laboratory of Soil and Sustainable Agriculture, Institute of Soil Sciences, Chinese Academy of Sciences, Nanjing, China; ^3^ College of Agriculture, Nanjing Agricultural University, Nanjing, China; ^4^ State Key Laboratory of Efficient Utilization of Arid and Semi-arid Arable Land in Northern China, Institute of Agricultural Resources and Regional Planning, Chinese Academy of Agricultural Sciences, Beijing, China

**Keywords:** air-filled porosity, artificial macropore, root-macropore interaction, soil structure, X-ray computed tomography

## Abstract

Maize is susceptible to hypoxia stress in soils with poor aeration, but the macropores have the potential to improve soil aeration. We studied the impact of artificial macropores on maize performance under poor aeration. Three levels of air-filled porosity (5%, 10% and 15%) were established, and soil columns with (28 vertical artificial macropores with 0.5 mm diameter) or without macropores were created for each level of air-filled porosity with a bulk density of 1.3 g cm^-3^. Root–macropore interactions were visualized using CT scanning (41 μm in resolution). Our results showed that root length density significantly increased by 114%, as air-filled porosity increased from 5% to 15%. However, when artificial macropores were present, an increase in air-filled porosity had no significant effect on root length density. The treatment of 5% air-filled porosity with macropores significantly increased root length density and root biomass by 108% and 65%, respectively, relative to the treatment of 5% air-filled porosity without macropores, whereas there was no significant difference in root growth between the treatments of 15% air-filled porosity with and without macropores. Compared to the treatment of 5% air-filled porosity with macropores, there was a significant reduction of 49% in the number of macropores colonized by roots under the treatment of 15% air-filled porosity with macropores. Our results demonstrate that macropores provide preferential paths for the colonization of maize roots, thereby promoting root growth under poor aeration. Creating macropores with bio-tillage can serve as a crucial strategy for enhancing crop performance in poorly aerated soils.

## Introduction

Soil aeration plays a crucial role in determining the oxygen content of soil, thereby significantly impacting the root development of crops (Ben-Noah and Friedman, 2018; Pedersen et al., 2020). Soil aeration is commonly assessed through the measurement of air-filled porosity, which is closely associated with soil water content (Wang et al., 2022). An increase in soil moisture content would result in a reduction in air-filled porosity, potentially resulting in insufficient oxygen supply when the air-filled porosity drops below 10%, thereby affecting the growth of crops (Xiong et al., 2020; da Silva et al., 1994). Zou et al. (2001) reported that the rate of root elongation tended to be close to zero when the air-filled porosity was less than 5%, whereas it increased significantly when the air-filled porosity was 15%. Furthermore, root growth in poorly aerated soil limited the diffusion of ethylene produced by roots, and the large accumulation of ethylene would hinder the growth of crop (Yamauchi et al., 2018; Vanhees et al., 2022; Pandey et al., 2021). Maize yield can be reduced by 50% under prolonged inadequate soil aeration conditions (Wang et al., 2021). Thus, it is imperative to implement effective measures to mitigate the deleterious impact of inadequate aeration on crop root growth.

Conventional tillage can enhance soil porosity and pore connectivity, thereby improving air permeability and diffusion in comparison to no-tillage or reduced-tillage in the short term (usually less than 2 years) (Pires et al., 2019; Schlüter et al., 2018; de Moraes et al., 2016). However, conventional tillage presents challenges in effectively enhancing soil aeration due to its high operational costs, short-lived effect, and susceptibility to secondary compaction (Busscher et al., 2002; Hamza and Anderson, 2008). Besides, conventional tillage presented potential disadvantages such as increased soil erosion, reduction of earthworms, loss of organic matter, disruption of soil structure over time (Chowaniak et al., 2020; Chan, 2001; Crittenden et al., 2015). Some studies have proposed using the deep and thick root systems of plants as a tillage tool to improve soil structure, and the macropores formed by root decomposition can effectively enhance soil aeration (Lucas et al., 2019; Chen et al., 2014; Zhang and Peng, 2021). Thus, utilizing macropores formed by crop roots in soils with limited air permeability may be a strategy to alleviate the negative effects of poor aeration on crop root growth.

The presence of macropores can greatly enhance the air permeability and oxygen concentration of subsoil, thereby facilitating crop root penetration into deeper soil layers for nutrient and water uptake (Colombi et al., 2017; Kautz, 2015). Several studies have reported that the artificial macropores in compacted soil (poor aeration and high strength) can enhance the total root length and root volume of maize (Xiong et al., 2022a; Pfeifer et al., 2014). However, the impact of macropores on root architecture under varying aeration levels induced by different soil water content is still unclear and requires further clarification.

The utilization of macropores by plant roots is closely linked to the physical condition of the soil. Xiong et al. (2022b) found that maize roots exhibited a preference for colonizing macropores in compacted soil but crossing macropores predominantly in uncompacted soil. This intriguing phenomenon was linked to soil strength, as excessively high mechanical resistance made it extremely challenging for roots to penetrate and necessitated entry into macropores (Atkinson et al., 2020). Furthermore, oxytropism (a behavior of roots growing toward high oxygen concentrations) might serve as an additional driving force for root growth toward macropores (Porterfield and Musgrave, 1998). Although previous studies have mentioned that oxygen or aeration might play a crucial role in root penetration into macropores, these experiments were conducted in compacted soil, making it challenging to establish the contribution of oxytropism (Pfeifer et al., 2014; Dexter, 1986; Colombi et al., 2017). Hence, the response of plant roots to macropores remains uncertain under poor aeration conditions.

Therefore, the aim of this study was to investigate the effect of macropores on maize performance, as well as the interactions between roots and macropores under poor aeration conditions. Our hypotheses were that (1) the artificial macropores promote crop growth under low air-filled porosity but not under high air-filled porosity and (2) the number of artificial macropores colonized by crop roots increase under poor aeration.

## Materials and methods

### Soil

The soil was collected from a wheat-maize cropping system located at Longkang farm, China (33° 32’ N, 115° 59’ E). The soil is classified as a Vertisol according to USDA soil taxonomy (Soil Survey Staff, 2003), and the soil texture is clay loam. The soil properties were as follows: pH 7.1, soil organic carbon 13.61 g kg^-1^, and total N 1.81 g kg^-1^ (Xiong et al., 2020). The proportions of sand (> 0.05 mm), silt (0.002-0.05 mm) and clay (< 0.002 mm) were 32.5%, 35.4% and 32.1%, respectively. The clay minerals were mainly composed of kaolinite, chlorite, hydromica, and montmorillonite, accounting for 29.0%, 23.3%, 23.0% and 13.7%, respectively (Xiong et al., 2020). To carry out the column experiment, the soil was air-dried and sieved to < 2 mm.

### Experimental design

In this study, the soil was packed with air-dried soil (< 2 mm) layer by layer (10 mm in each layer) in all columns (165 mm height × 75 mm diameter) until a height of 150 mm, filled with a soil bulk density of 1.3 g cm^-3^. The CT derived porosity of each soil column was consistent (**Supplementary Figure S1**). The columns were placed in water and saturated slowly by wetting 7 holes with a diameter of 3 mm in the bottom plate of the column (Xiong et al., 2022a). To investigate the impact of macropores on crop performance under various air-filled porosities, three sets of air-filled porosities (5%, 10% and 15%) were established, corresponding to volumetric water contents of 0.459, 0.409 and 0.359 cm ^3^ cm ^-3^, respectively. For each air-filled porosity treatment, 28 vertical artificial macropores were created throughout the whole soil column with a stainless steel needle with a diameter of 0.5 mm (0.14% macroporosity of these vertical pores), and treatments with no artificial macropores were used as the controls. Therefore, this experiment had six treatments: 5% air-filled porosity without macropores (5%), 5% air-filled porosity with macropores (5% + P), 10% air-filled porosity without macropores (10%), 10% air-filled porosity with macropores (10% + P), 15% air-filled porosity without macropores (15%), and 15% air-filled porosity with macropores (15% + P). There were three replicates in each treatment. The maize cultivar Zhengdan958 was selected, and the germinated seeds were placed 3 mm below the soil surface of each soil column for 15 days in September. In addition, plant height was recorded on the 6^th^, 11^th^, and 15^th^ days. Throughout the experiment, all the columns were weighed every day and sufficient water was added with a pipette to keep the soil moisture at its corresponding soil water content (Xiong et al., 2020).

### Scanning procedures

After 15 days of plant growth, the leaves and stems were cut off and dried in an oven at 80 °C to reach a constant weight and then weighed to obtain aboveground biomass (Xiong et al., 2020). Before scanning the soil, all the columns were wrapped in plastic film and placed in a refrigerator to prevent root shrinkage and soil moisture evaporation (Xiong et al., 2022a). The columns were scanned using an industrial X-ray μ-CT scanner (Phoenix Vtomex m, GE, Sensing and Inspection Technologies, GmbH, Wunstorf, Germany) in a multiscan mode set at 208 kV and 300 μA (Xiong et al., 2022b), which required approximately one hour to scan each column (Panel size was 400 × 400 mm) and the pixel/voxel resolution was 41 μm. Datos | × 2.0 software (GE, Sensing and Inspection Technologies, GmbH, Wunstorf, Germany) was used to reconstruct the projected images, and the number of projections was 1300. 16-bit grayscale CT slices were obtained in each column using the filtered back projection algorithm.

The soil was washed off of the roots on a 0.25 mm sieve after CT scanning was completed, and two-dimensional root characteristics (Root length density and root length at different diameters) were measured using a flatbed scanner (Epson Expression 1680) and WinRHIZO software (Regent Instrument Canada Inc.). The grayscale images were in 600 dpi resolution. The roots were dried in an oven at 80°C to measure the root biomass. The root length density was determined based on the total root length per soil volume.

### Image processing and analysis

The spatial interactions between artificial macropores and roots were analyzed using CT images. In this study, the root–macropore relationship was classified as “colonizing” or “no colonizing”, which meant that the roots continued to enter the macropores and did not exit from macropores immediately or that the roots crossed or did not enter the macropores, respectively. Following the method of Zhou et al. (2021); Atkinson et al. (2020), and Xiong et al. (2022b), the number of macropores colonized and not colonized by roots was counted manually from the CT images. Given the potential uncertainties associated with manual counting, meticulous records of all macropores were maintained and each slice was carefully scrutinized to ensure precise classification. In addition, the soil columns were broken to observe the growth of maize roots in macropores according to the method of Zhou et al. (2021). Pore connectivity was calculated using the method of Zhang et al. (2019).

### Penetration resistance

A microcomputer-controlled electronic universal testing machine (New Sansi Measurement Technology Co. LTD, Shenzhen, China) was used to measure the soil penetration resistance of each column under different air-filled porosities after CT scanning (Xiong et al., 2022a). In this study, a 2 mm diameter conical steel needle (30° full opening angle) was inserted vertically into the soil at a speed of 20 mm min^-1^ to a depth of 130 mm (Xiong et al., 2022a). Three locations of each soil column were randomly selected for the measurement.

### Statistical analysis

The impact of macropores on maize performance at the seedling stage under poor aeration was assessed by analysis of variance (ANOVA). The significant differences among the treatments at the *P* < 0.05 level were evaluated by one-way ANOVA with Duncan’s *post hoc* test. The interactions between root and macropore affected by air-filled porosity was analyzed using two-way ANOVA. The above statistical analyses were performed with the software IBM SPSS Statistics 22.

## Results

### Soil penetration resistance

**Figure 1:**
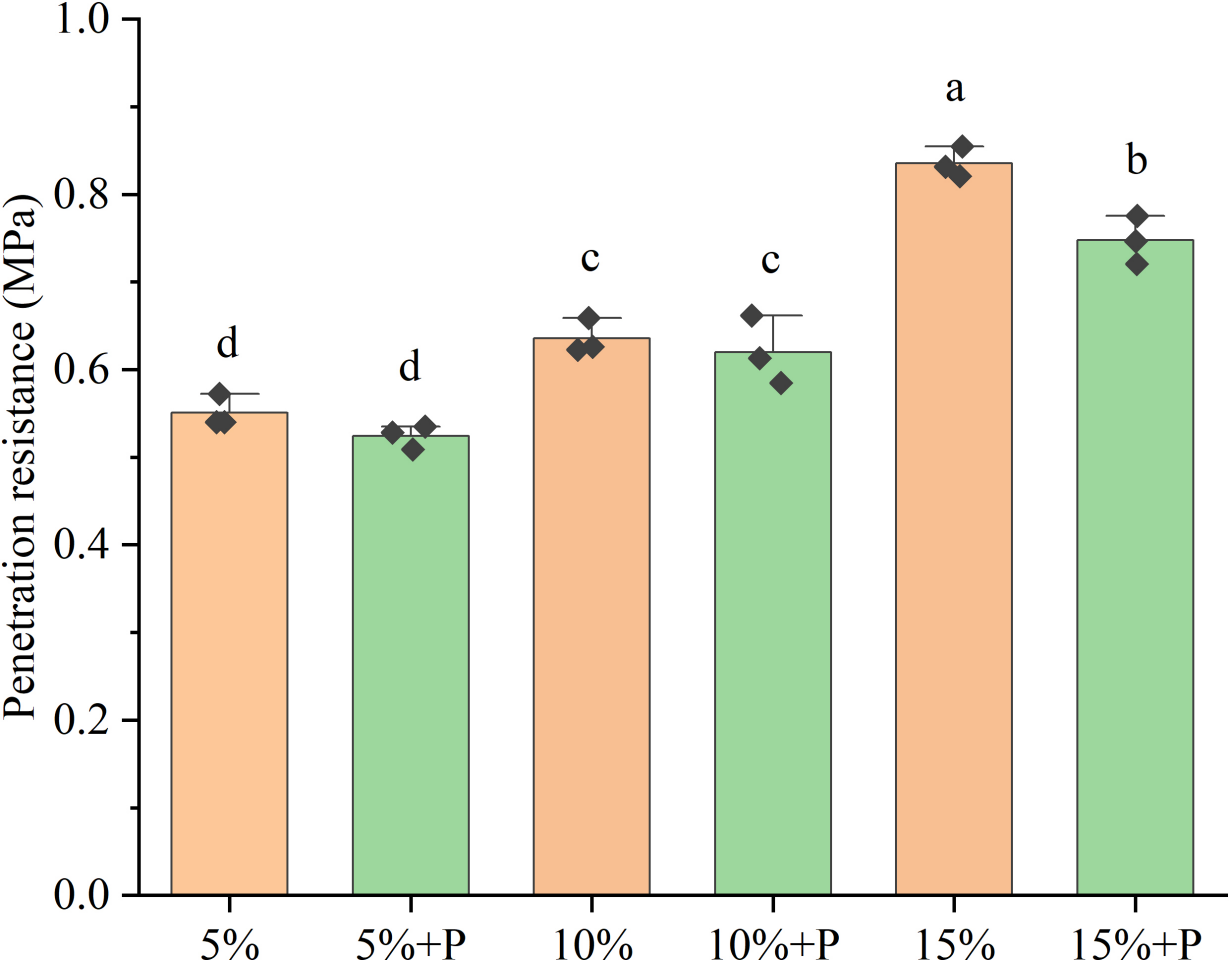
Penetration resistance of six treatments. Error bars are standard deviation of the mean (n = 3). Different lowercase letters above bars indicate that the means are significantly different (*P* < 0.05). 5%, 10% and 15% represent 5%, 10% and 15% air-filled porosity without macropores, respectively. 5% + P, 10% + P and 15% + P represent 5%, 10% and 15% air-filled porosity with macropores, respectively.

The soil penetration resistance increased with increasing air-filled porosity from 5% to 15%, by 52% without macropores and by 43% with macropores (**Figure 1**, *P* < 0.05). Relative to the treatments of 5% and 10% air-filled porosity without macropores (5% and 10% treatments), the presence of artificial macropores in these two treatments (5% + P and 10% + P treatments) had no significant impact on the soil penetration resistance (*P* > 0.05). However, the treatment of 15% air-filled porosity with macropores (15% + P treatment) significantly reduced the penetration resistance of soil by 11% compared to the treatment of 15% air-filled porosity without macropores (15% treatment) (*P* < 0.05).

### Shoot growth affected by air-filled porosity and macropores

**Figure 2:**
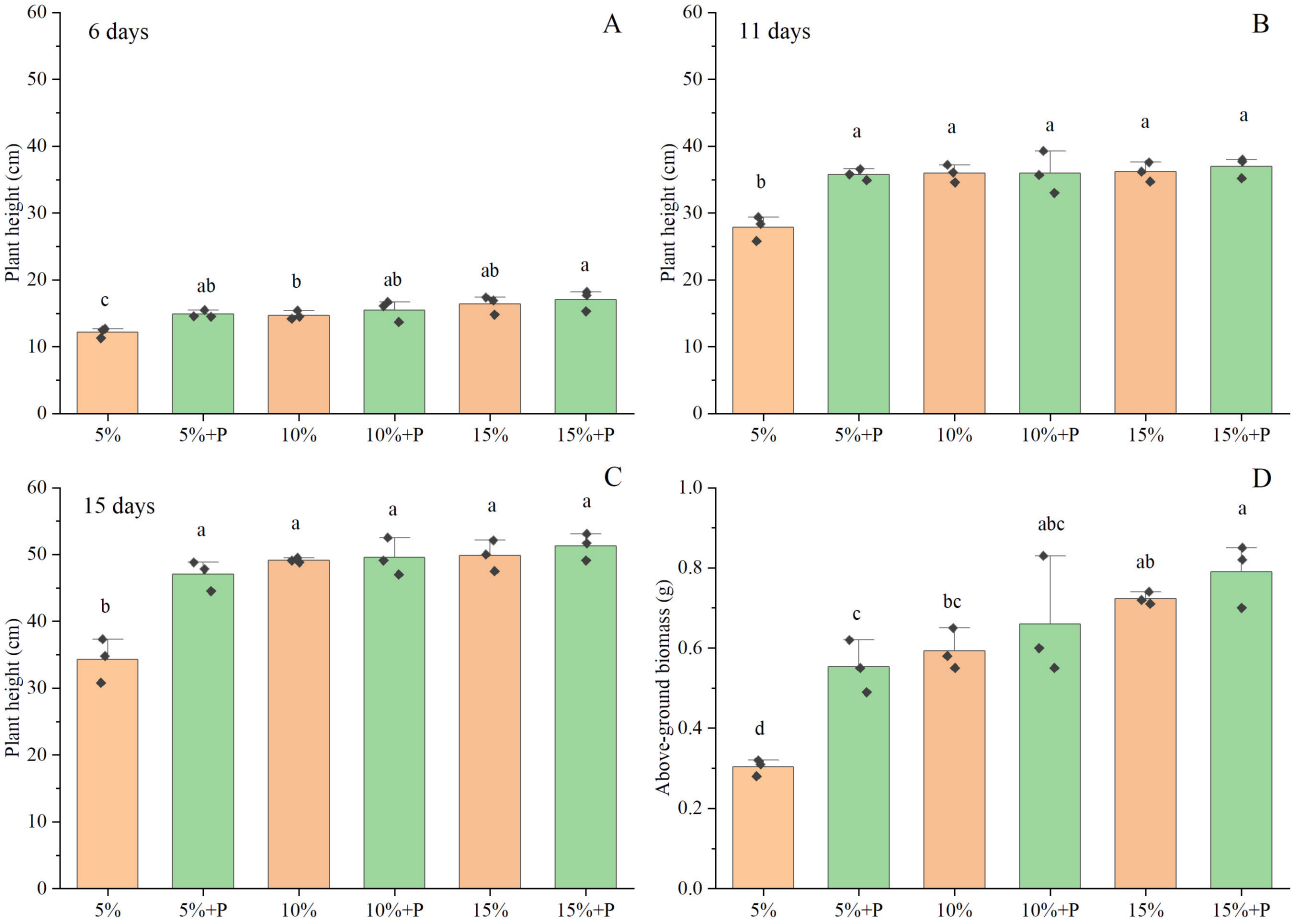
The effect of macropores on plant height **(A-C)** and above-ground biomass **(D)**. Error bars associated with histograms show standard deviation of the mean (n = 3). The different lowercase letters above bars indicate significant difference among the six treatments (*P* < 0.05). 5%, 10% and 15% represent 5%, 10% and 15% air-filled porosity without macropores, respectively. 5% + P, 10% + P and 15% + P represent 5%, 10% and 15% air-filled porosity with macropores, respectively.

The positive effects of air-filled porosity or macropores on the plant height of maize became more obvious on the 15^th^ day than on the 6^th^ and 11^th^ days (**Figures 2A-C**). Two-way ANOVA showed that both air-filled porosity and macropores significantly affected the shoot growth (*P* < 0.05; **Table 1**). The plant height and above-ground biomass increased with increasing air-filled porosity from 5% to 15%, by 45% and 138% in the absence of macropores (*P* < 0.05) and by 9% (*P* > 0.05) and 43% (*P* < 0.05) in the presence of macropores, respectively (**Figures 2C, D**). However, there was no significant difference in plant height and above-ground biomass between the treatments of 10% and 15% air-filled porosity, regardless of whether the macropores were present (*P* > 0.05). Compared to the treatment of 5% air-filled porosity without macropores (5% treatment), the artificial macropores significantly enhanced the plant height and above-ground biomass of maize by 37% and 82% under the treatment of 5% air-filled porosity with macropores (5% + P treatment), respectively (*P* < 0.05). However, the treatments of 10% and 15% air-filled porosity with artificial macropores (10% + P and 15% + P treatments) had little impact on the plant height and above-ground biomass of maize relative to the treatments of 10% and 15% air-filled porosity without macropores (10% and 15% treatments) (*P* > 0.05; **Figures 2C, D**).

**Table 1:** The two-way analysis of variance (ANOVA) of root traits and shoot growth affected by air-filled porosity and macropore.

Variation sources	Plant height (15 days)	Above-ground biomass	Root length density	Root biomass
Air-filled porosity	**0.000**	**0.000**	**0.000**	**0.024**
Macropore	**0.001**	**0.001**	**0.000**	**0.006**
Air-filled porosity × Macropore	**0.001**	**0.006**	**0.000**	0.337

The *P* values of the table is shown. Bold values indicate significant difference (*P* < 0.05).

### Root growth affected by air-filled porosity and macropores

**Figure 3:**
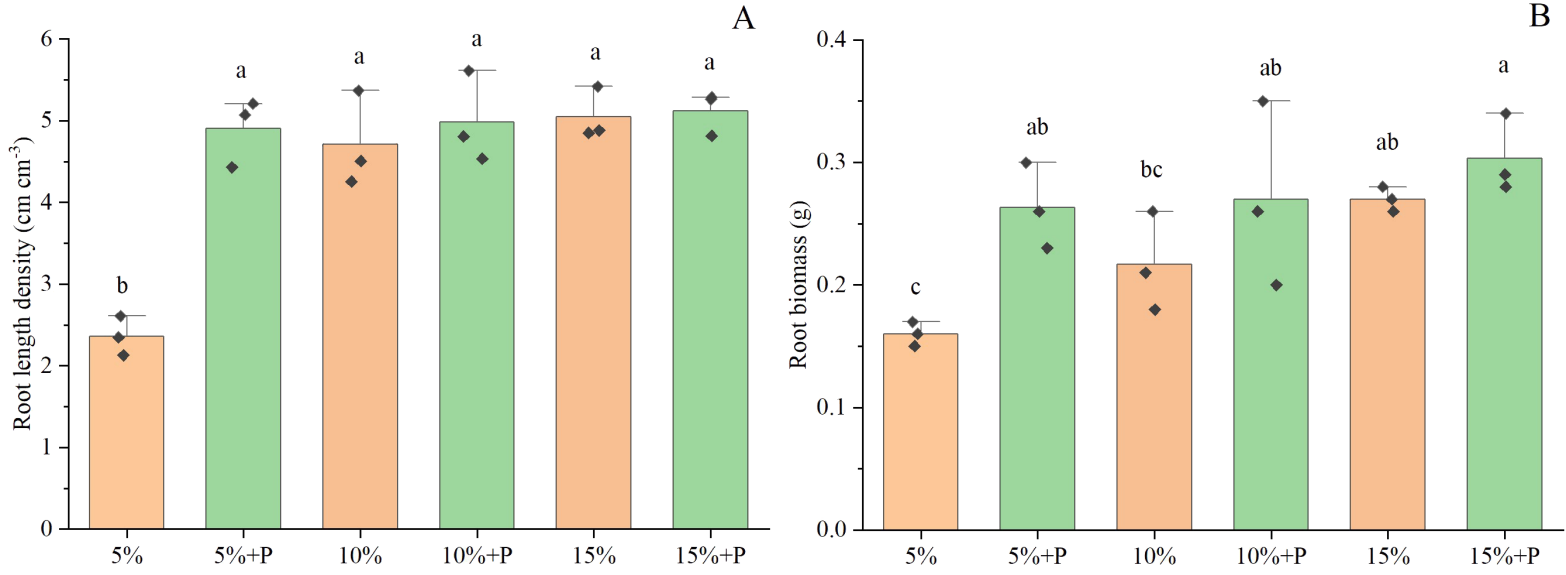
Root length density **(A)** and root biomass **(B)** of maize. Error bars associated with histograms show standard deviation of the mean (n = 3). The different lowercase letters above bars indicate significant difference among the six treatments (*P* < 0.05). 5%, 10% and 15% represent 5%, 10% and 15% air-filled porosity without macropores, respectively. 5% + P, 10% + P and 15% + P represent 5%, 10% and 15% air-filled porosity with macropores, respectively.

**Figure 4:**
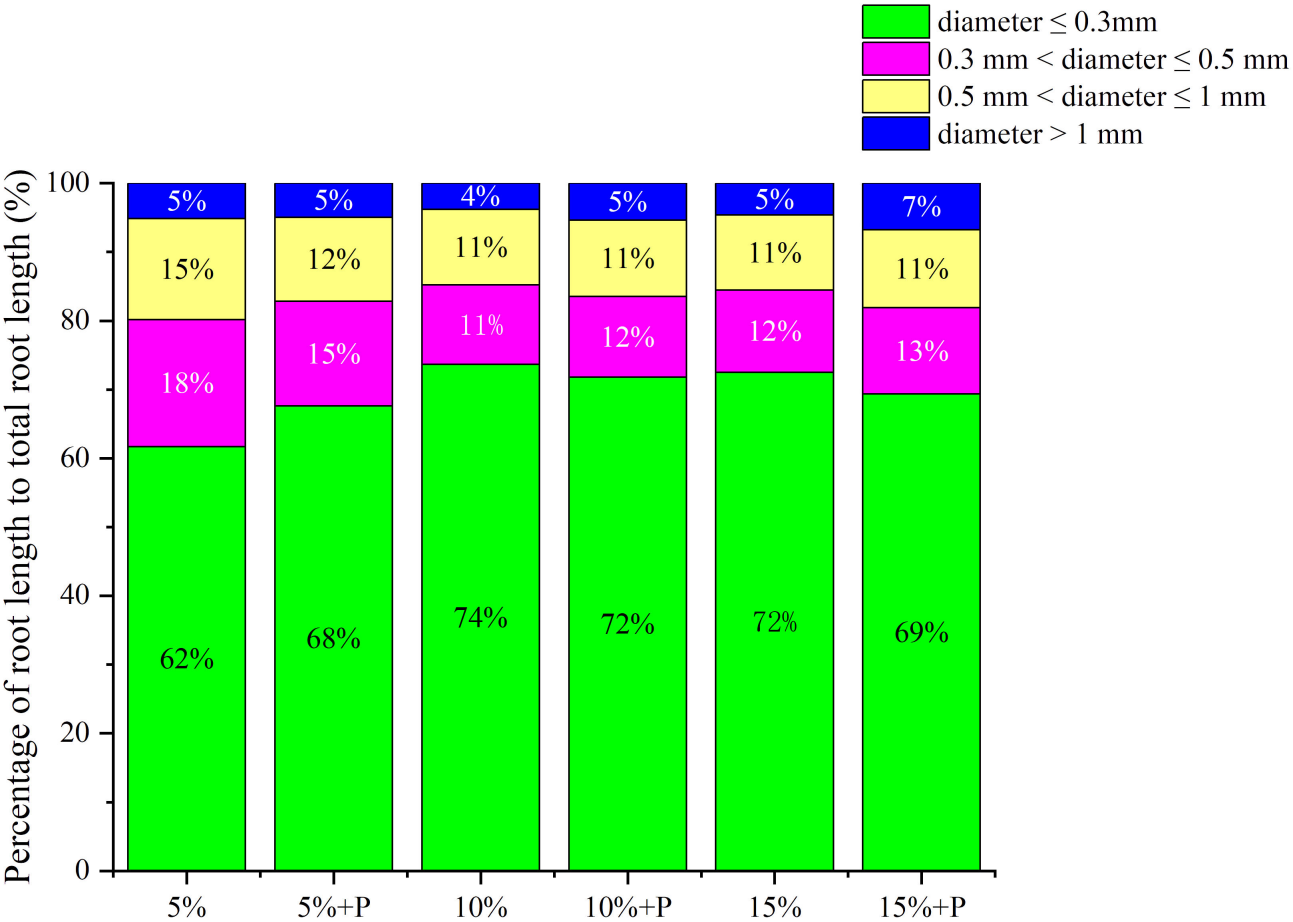
Percentage of root length to total root length in different root diameters. 5%, 10% and 15% represent 5%, 10% and 15% air-filled porosity without macropores, respectively. 5% + P, 10% + P and 15% + P represent 5%, 10% and 15% air-filled porosity with macropores, respectively.

Two-way ANOVA showed that both air-filled porosity and macropores significantly affected the root length density (**Table 1**). In soils without macropores, the root length density improved significantly by 114% with increasing air-filled porosity from 5% to 15% (*P* < 0.05). However, an increase in air-filled porosity had no significant effect on root length density in the treatments with macropores (*P* > 0.05) (**Figure 3**; **Table 1**). Relative to the treatment of 5% air-filled porosity without macropores (5% treatment), the presence of artificial macropores in the treatment of 5% air-filled porosity with macropores (5% + P treatment) significantly increased the root length density and root biomass by 108% and 65%, respectively (*P* < 0.05). However, compared to the treatments of 10% and 15% air-filled porosity without macropores (10% and 15% treatments), the artificial macropores had minor impacts on root length density and root biomass under the treatments of 10% and 15% air-filled porosity with macropores (10% + P and 15% + P treatments) (*P* > 0.05; **Figure 3**). Relative to the treatment of 5% air-filled porosity without macropores (5% treatment), the artificial macropores increased the length of roots with diameters less than 0.3 mm under the treatment of 5% air-filled porosity with macropores (5% + P treatment). However, the treatments of 10% and 15% air-filled porosity with macropores (10% + P and 15% + P treatments) did not increase the length of roots with diameters less than 0.3 mm compared to the treatments of 10% and 15% air-filled porosity without macropores (10% and 15% treatments) (**Figure 4**).

### Interactions between root and macropore affected by air-filled porosity

**Figure 5:**
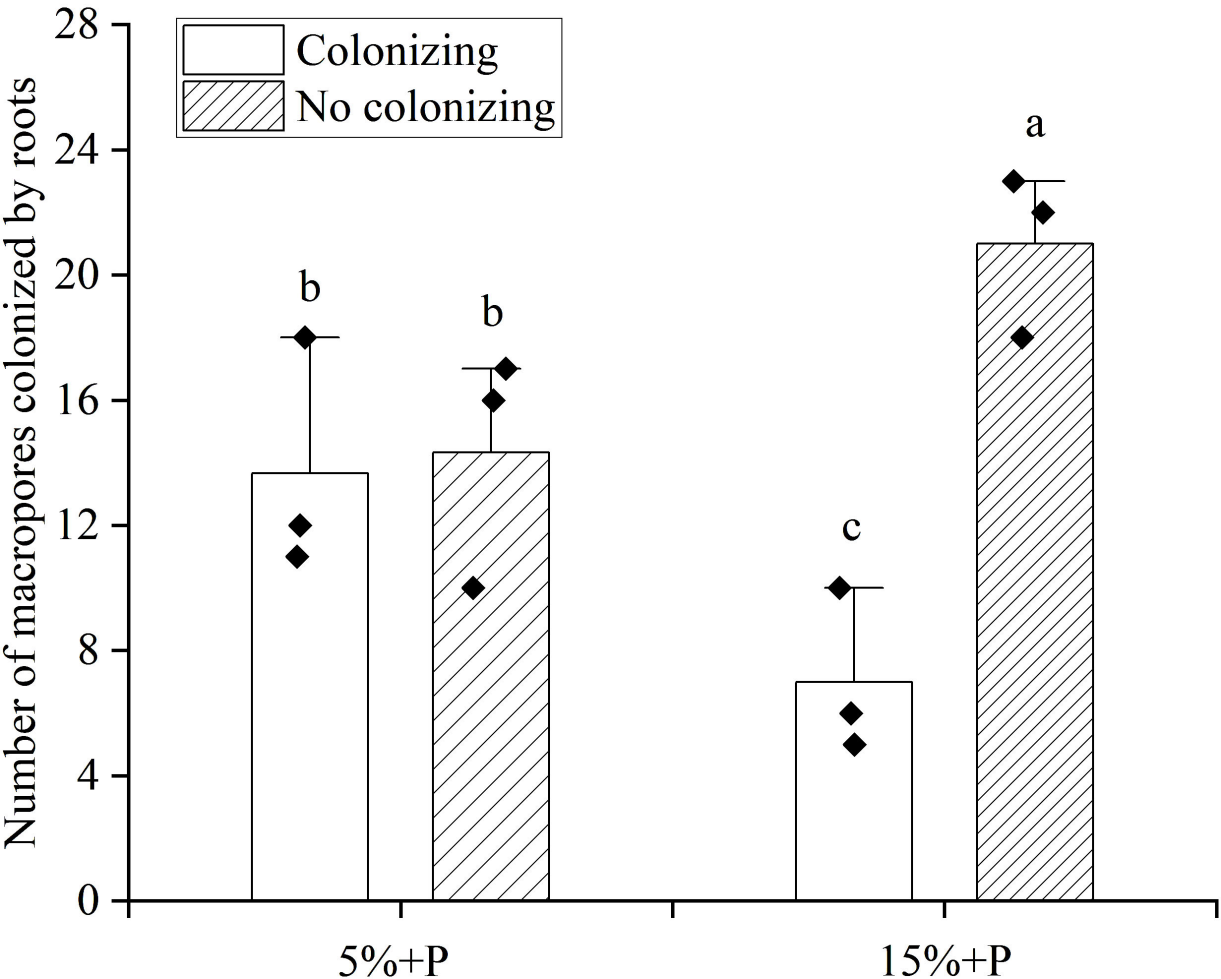
The number of macropores colonized by maize roots. Error bars associated with histograms show standard deviation of the mean (n = 3). Different lowercase letters above bars indicate significant differences of the means (*P* < 0.05). 5% + P and 15% + P represent 5% and 15% air-filled porosity with macropores, respectively.

The growth of roots in macropores (colonizing) could be clearly observed (**Supplementary Figure S2**). Compared with the treatment of 5% air-filled porosity with macropores (5% + P treatment), the number of artificial macropores colonized by maize roots was significantly reduced by 49% under the treatment of 15% air-filled porosity with macropores (15% + P treatment), whereas the number of artificial macropores not colonized by maize roots was significantly enhanced by 47% (*P* < 0.05). Furthermore, no significant difference was observed in the number of macropores colonized or not colonized by roots for the treatment of 5% air-filled porosity with macropores (5% + P treatment) (*P* > 0.05). However, the number of macropores not colonized by roots was 3 times higher than that colonized by roots for the treatment of 15% air-filled porosity with macropores (15% + P treatment) (*P* < 0.05; **Figure 5**).

## Discussion

### Influence of air-filled porosity on maize performance

The soil penetration resistance increased with increasing air-filled porosity (**Figure 1**), but it was much lower than critical value (2 MPa) of limiting crop growth (Bengough et al., 2011). Thus, the soil penetration resistance had a negligible impact on the growth of maize in this study. However, the air-filled porosity had a significant impact on maize growth. Our findings indicated that the above-ground biomass of maize increased significantly with an increase in air-filled porosity, regardless of whether the artificial macropores were present (**Figures 2C, D**). This result was consistent with the report by da Silva et al. (2004) that plant height was positively correlated with air-filled porosity. Uteau et al. (2015) found that low air-filled porosity (< 5%) resulted in low diffusion of oxygen to the root surface, which limited crop growth. In a recent study, Pandey et al. (2021) found that the reduction in air-filled pores and gas diffusion resulting from soil compaction led to the accumulation of ethylene in root tissue and triggered a hormonal response that limited plant growth. We inferred that the decreased plant height and above-ground biomass in the low air-filled porosity treatment might also be attributed to the accumulation of ethylene in root tissue due to low gas diffusion. Najeeb et al. (2015) reported that the waterlogging-induced hypoxia increased the accumulation of ethylene, which reduced the rate of leaf photosynthesis and stomatal conductance, thus limiting shoot growth of crops. In addition, high concentration of ethylene reduced stomatal conductance along with CO_2_ uptake, leading to a rapid decline in the availability of carbohydrates and soluble sugars, and the breakdown of starch and chlorophyll, which would further inhibit photosynthesis (Mohorović et al., 2024). The gas diffusion coefficient is generally enhanced with an increase in air-filled porosity, thereby facilitating the shoot growth of crops (Feng et al., 2002; da Silva et al., 2004). However, we found that the plant height and above-ground biomass of maize did not exhibit any significant difference between the 10% and 15% air-filled porosity treatments (**Figures 2C, D**). Sallam et al. (1984) found that the gas diffusion coefficient for 15% air-filled porosity was twice as high as that for 10% air-filled porosity and was 28 times higher than that of 5% air-filled porosity. Compared with 5% air-filled porosity, there was little difference in gas diffusion coefficients between 10% and 15% air-filled porosity. Thus, the reason might be attributed to the fact that approximately 9-12% of the air-filled porosity as reported by Uteau et al. (2015) was adequate for facilitating oxygen transportation needed for the rhizosphere. The 10% air-filled porosity was usually considered as the limit of soil aeration deficit above which the soil aeration was not a limitation for root growth (Valentine et al., 2012).

### Influence of macropores on maize performance under various air-filled porosities

Notably, the impact of air-filled porosity on maize root growth was intricately linked to the presence of macropores. We observed that an increase in air-filled porosity (5% to 15%) had a significant positive impact on root length density when macropores were absent, however, an increase in air-filled porosity had no significant effect on root length density in the treatments with macropores (5%+P to 15%+P) (**Figure 3**). The present study elucidated the interactive effects of air-filled porosity and macropores on root growth (**Table 1**). The availability of oxygen increased with the increase in air-filled porosity, which likely promoted the growth of maize roots (Liang et al., 1996). In soils with poor structure or low air permeability, the presence of macropores could significantly improve soil aeration (Colombi et al., 2017; Uteau et al., 2013; Mentges et al., 2016), which might weaken the effect of low air-filled porosity on root growth. Thus, increasing the number of macropores through the cultivation of cover crops with deep and robust root systems can be considered an effective strategy for alleviating poor soil aeration. Additionally, we found an increase in air-filled porosity in the macropore treatments facilitated the shoot growth of maize but had no significant impact on root growth (**Figures 2D**, **3**). This might be related to the high soil water content in our study, which had a positive effect on the accumulation of above-ground biomass (Koch et al., 2021).

In this study, the artificial macropores significantly improved maize growth under 5% air-filled porosity but not under 15% air-filled porosity (**Figures 2**, **3**). Our results confirmed the first hypothesis that the presence of macropores enhanced crop growth in soils with low soil aeration but not in those with high soil aeration. The excessive moisture content in the soil led to a decrease in gas diffusion rates and oxygen concentration levels (Abiko et al., 2012; Setter and Waters, 2003). Macropores have been demonstrated to enhance soil aeration, particularly in soils with limited air permeability (Uteau et al., 2022; Kautz, 2015). Although the insertion of a needle into the soil leaded to localized compaction around the artificial macropores, potentially affecting gas diffusion in our study, but this effect could be sufficiently counteracted as the artificial macropores significantly enhance the pore connectivity under the treatment of 5% air-filled porosity with macropores compared to the treatment of 5% air-filled porosity without macropores (**Supplementary Figure S3**). Furthermore, Vanhees et al. (2022) observed that exposure of maize to ethylene for extended periods of time slowed and might eventually stop the growth of axial roots. In the poor aeration, we speculated that the macropores also contributed to the diffusion of ethylene within the soil due to better pore connectivity and mitigated the adverse effects of excessive ethylene accumulation on plant root growth, as some roots could preferentially colonize the macropores. However, Stirzaker et al. (1996) found that the presence of large macropores with diameter of 3.2 mm in moist soils did not significantly increase the leaf area of barley. This discrepancy likely was attributed to the pore diameter, as the 0.5 mm diameter macropores used in our experiments resulted in good root-soil contact in the macropores and poor contact in the 3.2 mm macropores. Lucas et al. (2022) reported that different cover crops formed various macropore diameters due to differences in root architecture, with Saber Oat, Dwarf Essex Rapeseed, Annual Ryegrass, Oilseed Radish, and Austrian Winter Pea forming macropore diameters of 1.14, 1.23, 1.27, 1.39, and 1.40 mm, respectively. Han et al. (2015) found that chicory as a precrop increased root length of wheat compared to tall fescue, possibly because the macropore characteristics formed by chicory were more suitable for wheat growth. However, the roots significantly influenced the formation of macropores in the topsoil layer (15 cm), which decreased with increasing soil depth and disappeared at 30 and 50 cm soil layers (Stolze et al., 2022). Thus, we can choose different deep-rooted primer-plants to improve the soil structure and form appropriate size of macropores, which are more favorable to the root growth of following crops (Yunusa and Newton, 2003; Zhang and Peng, 2021).

Previous studies have reported that the plant roots tend to colonize macropores in compacted soil but cross them in non-compacted soil (Atkinson et al., 2020; Xiong et al., 2022b). The percentage of roots in the macropores increased from 30-40% to 85-100% as the soil depth increased from topsoil layers (< 60 cm) to subsoil layers (> 60 cm) (White and Kirkegaard, 2010). This indicated that plant roots could utilize macropores as a preferential pathway in high-strength subsoils or no-tillage system (Zhou et al., 2021; Ehlers et al., 1983). In this study, the number of macropores colonized by maize roots was found to be higher under the 5% air-filled porosity with macropores treatment than under the 15% air-filled porosity with macropores treatment (**Figure 5**). This result was revealed for the first time and supported the second hypothesis that the colonization of artificial macropores by maize roots increased in response to poor soil aeration conditions. The presence of macropores could provide a fast path with high oxygen content for crop root growth (Xiong et al., 2022c; Colombi et al., 2017). We inferred that when the air-filled porosity was as low as 5%, the roots had to grow into the macropores to access additional oxygen to mitigate aeration stress. Conversely, once the air-filled porosity reached 15%, there was an ample supply of oxygen in the soil for root respiration, thereby eliminating the necessity for growth toward macropores. Therefore, the macropores might act as a shelter for the roots when they encounter stresses from poor soil conditions, while their role in root growth might be minor in favorable soil conditions (Landl et al., 2019).

### Limitation of air-filled porosity as an indicator for soil aeration

The index of air-filled porosity has been widely utilized in various studies to evaluate soil aeration (da Silva et al., 1994; Kreba et al., 2017; Wang et al., 2022). Generally, soil aeration was considered poor when the air-filled porosity fell below 10%, which could restrict crop growth (da Silva et al., 1994). In this study, the presence of macropores significantly enhanced the root length density and root biomass of maize in the 5% air-filled porosity treatment, which were similar to those in the 15% air-filled porosity treatment (**Figure 3**). In other words, the macropores might increase the soil aeration in the 5% air-filled porosity treatment to the level of the 15% air-filled treatment for maize root growth. However, the porosity of the macropores was only 0.14% in this study, which accounted for only a very small part of the soil volume but played an important role in improving soil aeration. This was attributed to greater connectivity of the macropores than the disconnected non-macropores (packing voids). Giuliani et al. (2024) reported that the evaluation of soil aeration should incorporate pore diameter and connectivity in addition to focusing on the threshold of air-filled porosity. Zhang et al. (2018) found that the pore connectivity of biopores was 8 times higher than that of non-biopores in upland subsoil on average. Colombi et al. (2017) observed that the relative gas diffusion coefficient at a soil depth of 30 cm in compacted soil containing macropores was 32 times greater than that in non-macropore-containing compacted soil and 1.8 times greater than that in uncompacted soil, resulting in a higher oxygen content in macropores. Therefore, in cases where the air-filled porosity was very low, the presence of macropores in the soil might prevent the occurrence of anoxic conditions. In summary, it might not be accurate to use only the index of air-filled porosity to measure soil aeration when macropores are present.

## Conclusions

Whether macropores existed or not, the above-ground biomass of maize were enhanced with increasing air-filled porosity, but the influence of air-filled porosity on root growth was intimately associated with macropores. In the absence of macropores, an increase in air-filled porosity enhanced root length density, while the presence of macropores led to no significant effect of air-filled porosity on root growth. The presence of macropores significantly enhanced shoot and root traits under the 5% air-filled porosity treatment, whereas their impact on these parameters was negligible under the 15% air-filled porosity treatment. The macropores facilitated maize root colonization within the macropores and stimulated the elongation of roots under conditions of poor soil aeration. However, the enhancement of air-filled porosity led to a significant reduction in the number of artificial macropores colonized by maize roots, resulting in no significant effect of macropores on maize performance. This study has demonstrated the beneficial impact of macropores on maize shoot and root growth in poorly aerated conditions, and creating macropores can effectively improve maize performance at the seedling stage under poor aeration.

## Data Availability

The original contributions presented in the study are included in the article/**Supplementary Material**. Further inquiries can be directed to the corresponding authors.
