# Supplementary material for: Artificial macropores improve maize performance at the seedling stage under poor aeration
Source: Front Plant Sci. 2024 Oct 3;15:1468242. doi: 10.3389/fpls.2024.1468242 (PMC11483996; doi:10.3389/fpls.2024.1468242)
Supplement: Supplementary file 1 [file Image1.pdf]

Figure captions

Fig. S1. The CT derived porosity throughout the soil column. Error bars associated with histograms show standard deviation of the mean (n = 3). Different lowercase letters above bars indicate significant differences of the means ($P < 0.05$). 5% + P and 15% + P represent 5% and 15% air-filled porosity with macropores, respectively.

Fig. S2. Image of the growth of maize roots in macropores.

Fig. S3. Pore connectivity between the treatments of 5% air-filled porosity with and without macropores. Error bars associated with histograms show standard deviation of the mean (n = 3). Different lowercase letters above bars indicate significant differences of the means ($P < 0.05$). 5% and 5% + P represent 5% air-filled porosity without macropores and with macropores, respectively.

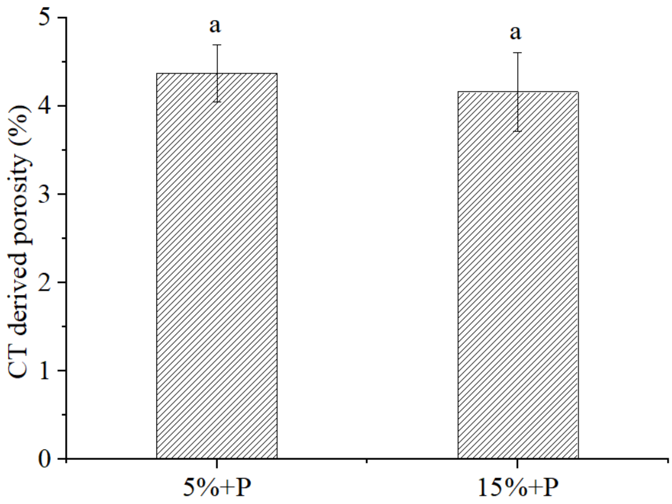

Fig. S1. The CT derived porosity throughout the soil column. Error bars associated with histograms show standard deviation of the mean (n = 3). Different lowercase letters above bars indicate significant differences of the means ($P < 0.05$). 5% + P and 15% + P represent 5% and 15% air-filled porosity with macropores, respectively.

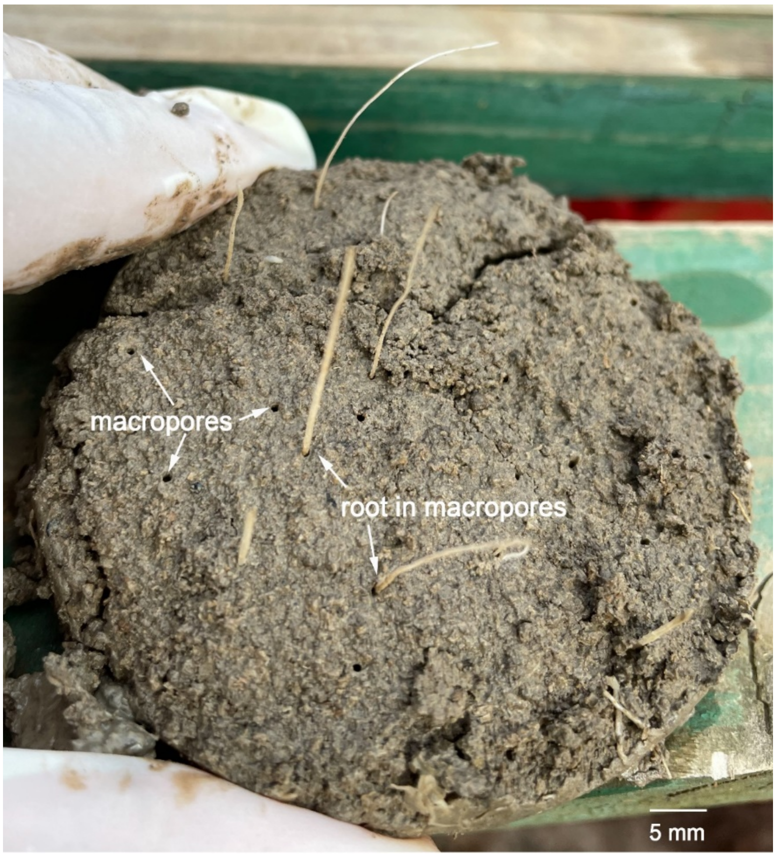

Fig. S2. Image of the growth of maize roots in macropores.

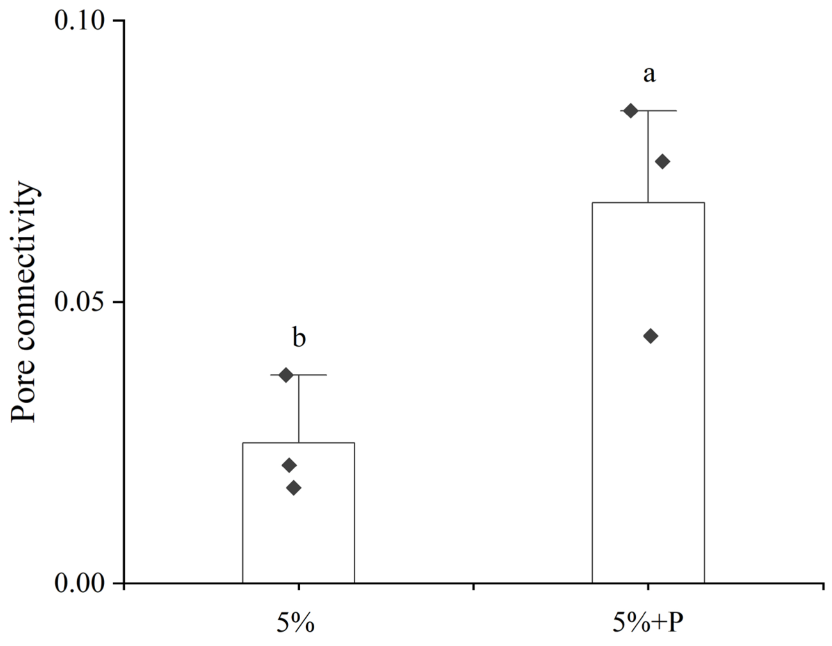

Fig. S3. Pore connectivity between the treatments of 5% air-filled porosity with and without macropores. Error bars associated with histograms show standard deviation of the mean (n = 3). Different lowercase letters above bars indicate significant differences of the means ($P < 0.05$). 5% and 5% + P represent 5% air-filled porosity without macropores and with macropores, respectively.
